# Effect of nitrogen compounds on transport of ruthenium through the RCS

**DOI:** 10.1007/s10967-017-5172-7

**Published:** 2017-01-11

**Authors:** Ivan Kajan, Teemu Kärkelä, Ari Auvinen, Christian Ekberg

**Affiliations:** 1 Chalmers University of Technology, 41296 Göteborg, Sweden; 2 VTT Technical Research Centre of Finland Ltd, 02044 Espoo, Finland

**Keywords:** Ruthenium, Ruthenium tetroxide, Nuclear accidents, Primary circuit, Air radiolysis

## Abstract

Ruthenium is a fission product that can be released from the fuel in case of a severe nuclear accident. In this work the impact of the atmosphere composition, including air radiolysis products, on the transport of ruthenium through a primary circuit was examined. Experiments were performed at temperatures 1300, 1500 and 1700 K in a slightly humid air. In the experiments significant effect of nitrogen oxides (N_2_O, NO_2_) and nitric acid on the ruthenium chemistry in the model primary circuit was observed. The obtained results indicate a strong effect of air radiolysis products on the quantity partitioning of transported ruthenium to gaseous and aerosol compounds.

## Introduction

In a nuclear accident the main concern is that elements prone to form volatile compounds will be released from the fuel. Due to the ability of ruthenium to form volatile oxides and radiological risk via isotopes ^103^Ru and ^106^Ru, ruthenium is concerned as one of the critical elements in the case of a nuclear accident.

**Fig. 1:**
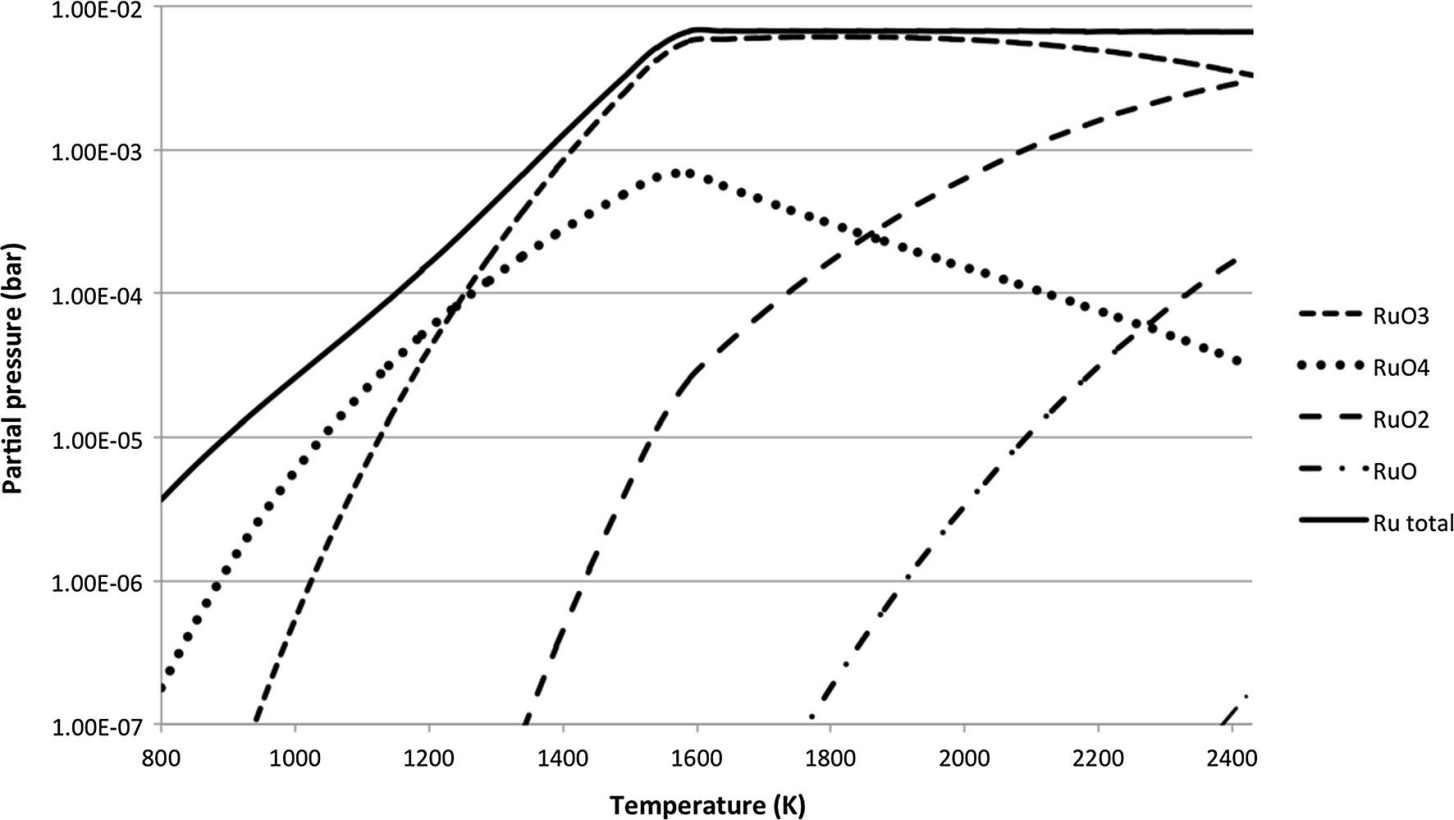
Ruthenium gaseous species at thermodynamic equilibrium in air atmosphere at 1 bar pressure [4]

Proper quantification of the release and transport rates of radionuclides is necessary to evaluate the possible source term as accurately as possible. Consequent interactions of these nuclides with surface materials within the containment and other fission products can be then evaluated. The release of fission products from the irradiated nuclear fuel samples under different experimental conditions was investigated during the PHÉBUS FP and VERCORS research programs [1–3]. Under these integral experiments releases of ruthenium were significant when up to 17% of ruthenium content was released from the fuel [1]. A strong dependence of the ruthenium release rates on the oxygen content and temperature was observed [2, 3]. The thermodynamic equilibrium composition of ruthenium gaseous species calculated by the Factsage thermochemical software predicts the main volatile oxides to be RuO_2_, RuO_3_ and RuO_4_ depending on temperature, as shown in Fig. 1 [4].

Both gaseous RuO_2_ and RuO_3_ are unstable at lower temperatures, which makes their partial pressures in the gaseous atmosphere very low at temperatures below 1000 K [5]. RuO_2_ condenses and RuO_3_ decomposes into the form of solid RuO_2_ according to Eqs. (1) and (2), where *K* is the equilibrium constant [5]. Although RuO_4_ is also not stable at the low temperatures, its decomposition kinetics are much slower when compared to RuO_3_(g) and RuO_2_(g) [6]. Thus RuO_4_ is the most relevant form in the conditions where temperatures lower than 1000 K are expected.

1 $${\text{RuO}}_{2}({\text{g}}) \to {\text{RuO}}_{2}({\text{s}}) \quad {K} = 5{\text{E}}12\;{\text{at}}\;1000\;{\text{K}},$$

2 $$2 {\text{RuO}}_{3}({\text{g}}) \to 2{\text{RuO}}_{2}({\text{s}}) + {\text{O}}_{2}({\text{g}}) \quad {K} = 3.7{\text{E}}12\;{\text{at}}\;1000\;{\text{K}}.$$

Very few studies deal with the transport of ruthenium through the primary circuit of a nuclear power plant [7–9]. The humidity, temperature and the flow rate of air-flow have been shown to be the main factors affecting the ruthenium transport in the primary circuit conditions. The transported ruthenium was observed to be in the form of aerosols consisting of RuO_2_ and in the form of gaseous RuO_4_. The quantity and the chemical form of transported ruthenium can also be affected by interactions with other elements released from the fuel [10]. Thus using the pristine air atmosphere in the experiments seems to be oversimplified in the case of severe accident studies due to the occurrence of aerosols (e.g., fission products, control rod materials) as well as various gaseous compounds, such as produced by the radiolysis of air [1, 11]. Experimental data is lacking, however, for atmospheric compositions other than dry or humid air. Only a few studies have addressed the effect of gaseous and particulate additives on the ruthenium chemistry in air–steam atmospheres [10, 12–15].

During a nuclear accident the main air radiolysis products expected in the containment atmosphere are ozone and nitrogen oxides such as NO_2_ and N_2_O. When humid atmosphere is taken into account the reaction of NO_2_ with water leads to the production of HNO_3_. All these compounds show oxidizing properties [16] and thus can oxidize lower ruthenium oxides into the form of RuO_4_ in the primary circuit of a nuclear power plant. The proposed reactions of RuO_2_ and RuO_3_ oxidation to RuO_4_ are presented in Eqs. (3)–(8) together with the corresponding equilibrium constants as calculated by HSC 5.11 chemistry software [17].

3 $${\text{RuO}}_{3}({\text{g}}) + {\text{NO}}_{2}({\text{g}}) \leftrightarrow {\text{RuO}}_{4}({\text{g}}) + {\text{NO}}_{{}}({\text{g}}) \quad {K} = 16.8\;{\text{at}}\;1500\;{\text{K}},$$

4 $${\text{RuO}}_{3}({\text{g}}) + 2{\text{N}}_{2} {\text{O}}({\text{g}}) \leftrightarrow {\text{RuO}}_{4}({\text{g}}) + 2{\text{N}}_{2} ({\text{g}})\quad {K} = 9.5{\text{E}}5\;{\text{at}}\;1500\;{\text{K}},$$

5 $$3{\text{RuO}}_{3}({\text{g}}) + 2{\text{HNO}}_{3}({\text{g}}) \leftrightarrow 3{\text{RuO}}_{4}({\text{g}}) + 2{\text{NO}}({\text{g}}) + {\text{H}}_{2} {\text{O}}({\text{g}})\quad {K} = 4.5{\text{E}}10\;{\text{at}}\;1500\;{\text{K}},$$

6 $${\text{RuO}}_{2}({\text{s}}) + 2{\text{NO}}_{2}({\text{g}}) \leftrightarrow {\text{RuO}}_{4}({\text{g}}) + 2{\text{NO}}({\text{g}})\quad {K} = 2.8\;{\text{at}}\;1500\;{\text{K}},$$

7 $${\text{RuO}}_{2}({\text{s}}) + 2{\text{N}}_{2} {\text{O}}({\text{g}}) \leftrightarrow {\text{RuO}}_{4}({\text{g}}) + 2{\text{N}}_{2}({\text{g}})\quad {K} = 9.0{\text{E}}9\;{\text{at}}\;1500\;{\text{K}},$$

8 $$1.5{\text{RuO}}_{2}({\text{g}}) + 2{\text{HNO}}_{3}({\text{g}}) \leftrightarrow 1.5{\text{RuO}}_{4}({\text{g}}) + 2{\text{NO}}({\text{g}}) + {\text{H}}_{2} {\text{O}}({\text{g}})\quad {K} = 4.5{\text{E}}7\;{\text{at}}\;1500\;{\text{K}}.$$

To provide a more precise and realistic modelling of the ruthenium chemistry in the primary circuit conditions the interaction of aerosols and air radiolysis products with Ru oxides in the gas phase needs to be evaluated. Therefore, to have a better insight into the chemistry of ruthenium during its transport through the RCS, the effects of nitrogen compounds (NO_2_, N_2_O, HNO_3_) on the transport and speciation of ruthenium were examined in this work.

## Experimental

### Experimental facility and procedure

**Fig. 2:**
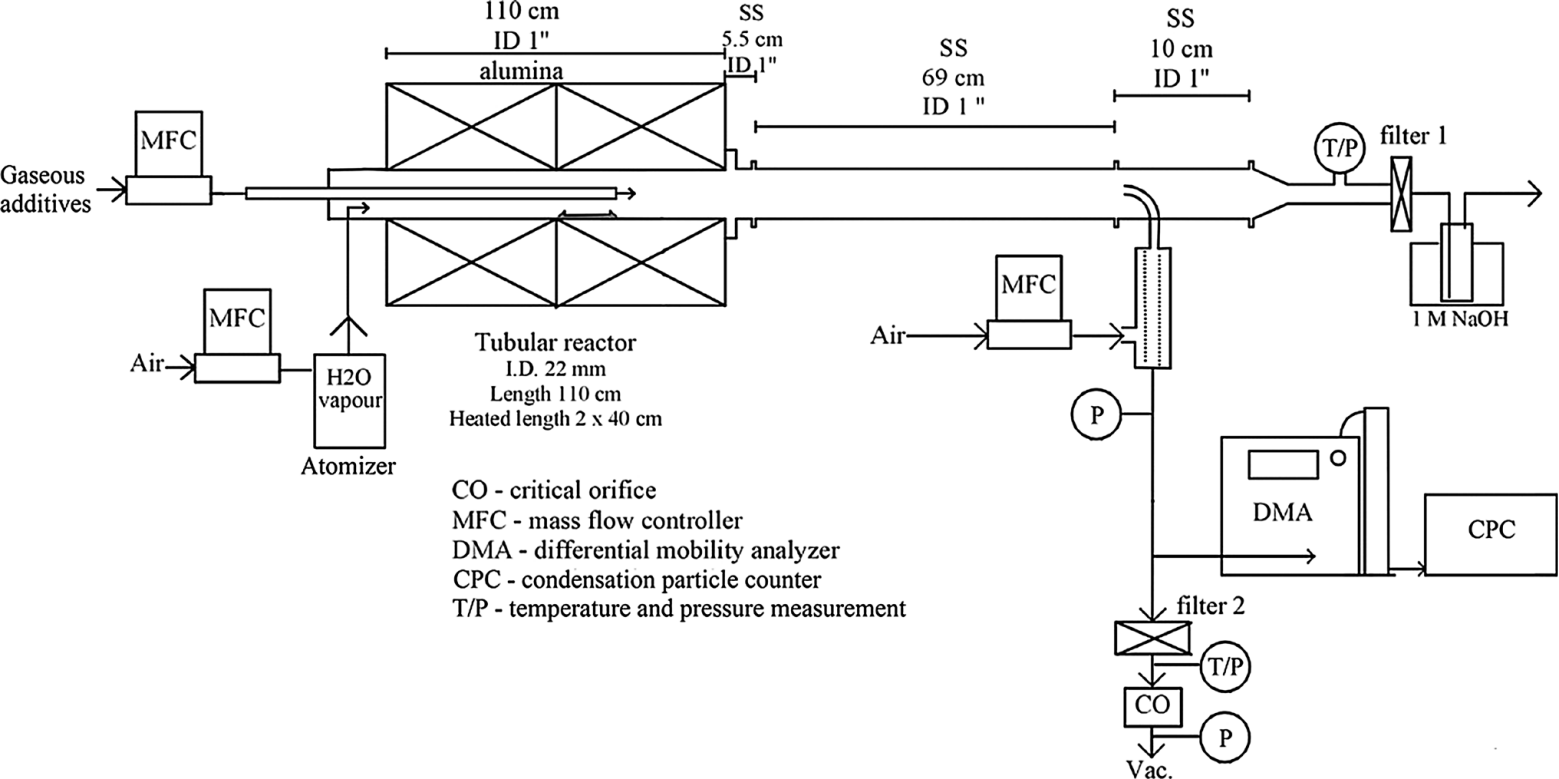
Schematics of the experimental facility for ruthenium transport studies

The configuration of the “VTT’s Ru transport facility” for the experiments is presented in Fig. 2. A detailed description of the facility is provided in previous work [7, 13, 15]. The main component of the facility was the horizontal, tubular flow furnace (Entech, ETF20/18-II-L). This was used to heat the anhydrous RuO_2_ powder (99.95%, Alfa Aesar). The furnace was 110 cm long and had two heating sections, each 40 cm long. These zones were separated by a 38 mm layer of insulation. At both ends of the furnace there was 131 mm of thermal insulation. The furnace tube was made of high purity alumina (Al_2_O_3_, 99.7%) and its inner diameter was 22 mm. The alumina crucible (length 20 cm) with the RuO_2_ powder (mass 1 or 2 g depending on the temperature used in the experiment) was placed at the beginning of the second heated zone of the furnace. As a new feature in these experiments a second alumina tube (Al_2_O_3_, 99.7%, outer diameter 6 mm with a wall thickness of 1 mm) was inserted inside the furnace tube, with the outlet located directly after the crucible. The RuO_2_ powder was heated to 1300, 1500 or 1700 K in an oxidizing flow in order to produce gaseous ruthenium oxides.

The total flow rate through the facility was 5.0 ± 0.1 l/min (NTP; conditions 0 °C, 101,325 Pa, measured using a thermal mass flowmeter TSI 3063, TSI Incorp.). Half of the total flow was directed through the inner furnace tube and the rest of the flow passed through the furnace tube. The pressure inside the facility ranged from 102 to 104 kPa. The air flow (2.5 ± 0.1 l/min, NTP) directed to the furnace tube was fed through an atomizer (TSI 3076). The air flow transported the water droplets (Milli-Q, ultrapure water, resistivity of 18.2 MΩ cm at 25 °C) produced by atomizer via the heated line (120 °C) into the inlet of the furnace. Water evaporated when the droplets were heated and therefore led to an increase in the steam concentration within the furnace. A flow of N_2_O, NO_2_ or HNO_3_ gases (2.5 ± 0.1 l/min, NTP) was fed through the inner furnace tube. NO_2_ was diluted with N_2_ to obtain a similar concentration of precursor as in the case of N_2_O. As HNO_3_ was fed using an additional atomizer located before the inlet of inner furnace tube (not shown in Fig. 2), a carrier gas of nitrogen was used to transport HNO_3_ droplets (solution of HNO_3_ and Milli-Q water) via the heated line (120 °C) into the inlet of the inner furnace tube. The experimental matrix is presented in Table 1. The duration of experiments was 60 min for the experiments conducted in the humid air atmosphere (experiments 1–3) and 20 min for the experiments with additive precursors fed into the humid air atmosphere (experiments 4–12).

**Table 1:** Detailed experimental matrix

*T* (K)	Gas	Flow rate over the crucible (l/min)^a^	Precursor^b^	Additive precursor concentration	Humidity^c^ (ppmV)	Other
1300 ± 12 1500 ± 12 1700 ± 12	Air	2.5	RuO_2_	–	2.14E+04 ± 2.1E3	Atomizer with water only
1300 ± 12 1500 ± 12 1700 ± 12	Air + NO_2_	2.5	RuO_2_ + NO_2_	NO_2_ 50 ppmV	2.14E+04 ± 2.1E3	Atomizer with water only
1300 ± 12 1500 ± 12 1700 ± 12	Air + N_2_O	2.5	RuO_2_ + N_2_O	N_2_O 50 ppmV	2.14E+04 ± 2.1E3	Atomizer with water only
1300 ± 12 1500 ± 12 1700 ± 12	Air + HNO_3_	2.5	RuO_2_ + HNO_3_	HNO_3_ 5 ppmV	8.3E+04 ± 8.3E3	Atomizer with HNO_3_ solution

^a^The total flow rate through the furnace over the crucible was 2.5 ± 0.1 l/min (NTP) before the inner tube outlet and 5 ± 0.1 l/min (NTP) after the inner tube outlet in every experiment

^b^The mass of RuO_2_ powder in the crucible was 1 g for temperatures 1300 and 1500 K and 2 g for temperature 1700 K

^c^The humidity in the gas flow came from the water-based precursor solution of the atomizer. The increase of humidity in the HNO_3_ experiments is due to water evaporation from the HNO_3_ solution injected into the inner tube

After the vaporization of Ru and the following reactions within the gaseous atmosphere, the gaseous and particulate reaction products were trapped in a NaOH solution and collected on planer filters, respectively. Further details of this are provided in a previous work [13]. Particles were also analyzed online (see details below).

### Analysis methods

#### Ruthenium release

The release rate of ruthenium in the experiments was obtained by weighing the mass of the crucible containing RuO_2_ before and after the experiments. The mass of released RuO_2_ was then converted to the corresponding mass of elemental ruthenium. Based on the previous study performed with the same facility using ^103^Ru radiotracer [7], the release of ruthenium from the crucible was assumed to be linear during the experiments.

#### Online analysis of ruthenium transport

The number size distribution of particles was measured online with a combination of a differential mobility analyser (DMA, TSI 3080/3081) and a condensation particle counter (CPC, TSI 3775) with a time resolution of 3 min. The flow rate through these devices was 0.30 ± 0.01 l/min (NTP). The particles were size classified according to their electrical mobility by the DMA and the number of particles in each size class was counted by the CPC (with a counting efficiency higher than 96%). The measurement range was from 15 to 670 nm. However, a pre-impactor removed particles larger than 615 nm at the inlet of the DMA. The measurement system was controlled with the Aerosol Instrument Manager software version 9.0 (TSI). This measurement system is known as a scanning mobility particle sizer (SMPS).

All the online measurement data presented was corrected by considering the loading of the analysis filter by particles and the following decrease in the flow rate through the filter, and therefore took into consideration the decreased flow rate into the aerosol sampling line from the main line. Correction was based on the calibration of flow rate through the critical orifice (CO) at various temperatures and pressures, simulating the loading of the filter. The calibration data was then used to estimate the flow rate of CO in the experiments, with the help of temperature and pressure measurement data. The flow rate from the main line to the aerosol line was also always measured with a thermal mass flowmeter at the beginning of every experiment. As a result, the changes in dilution ratio could be taken into consideration. The highest uncertainty in the dilution ratio originated from the inaccuracy of the mass flow controller feeding air through the porous tube dilutor and the thermal mass flowmeter. Given that the uncertainty of both devices can be ±2% of the reading, the uncertainty in the dilution ratio was ca. ±4%. Otherwise the contribution of uncertainties in temperature and pressure measurements to the dilution ratio was low, since the flow rate through the CO did not vary significantly due to these uncertainties. The online data presented was also dependent on the flow rate through the main line. The flow rate was always measured at the beginning of the experiments, and an additional uncertainty of ±2% resulted from the flowmeter. Therefore, the combined conservative uncertainty estimate for the online data presented was ca. ±6%. The particle number concentration values measured with CPC could also be too low by up to 4% due to the deficiency in counting efficiency.

#### Instrumental neutron activation analysis (INAA)

The quantification of ruthenium aerosols collected on filters and gaseous ruthenium trapped in the sodium hydroxide liquid traps was carried out by INAA. Ruthenium in the liquid traps was precipitated with addition of EtOH (96% Sigma-Aldrich), centrifuged and then filtered from the solution. Aerosols collected on the PTFE filters were used as they were after the experiment. Samples were then irradiated in the research reactor at VTT (Triga mark II reactor in Otaniemi, Espoo). Irradiations were performed with a thermal neutron flux of 8.7 × 10^12^ n/cm^2^/s and epithermal flux of 4.6 × 10^12^ n/cm^2^/s. Samples were irradiated for periods of from 10 min up to 4 h, depending on the ruthenium content in the sample. After 1 week of cooling time, the samples were measured by means of gamma spectrometry.

For these measurements a high purity germanium detector (Ortec model GEM-15180-S) was used with a relative efficiency of 17.7% and resolutions of 1.7 keV at 1332 keV. The evaluation of data was carried out using GammaVision software version 7.01.03. (Ortec). The detector was empirically calibrated for both energy and efficiency with QCYA18189 (Eckert and Ziegler) standard radionuclide source solution with the same geometry as irradiated samples.

The activity of ^103^Ru was determined from counts at the 497 keV peak, where absolute efficiency at a given geometry was determined to be 1.7%. The detection limit for ruthenium was determined to be 1.0E−2 µg based on the times of irradiations and measurements. Cross sections used for the data evaluation were taken from a previous publication [18]. Uncertainty of the measurements was calculated to be 5% according to the guide to the expression of uncertainties in measurements [19].

### Chemical characterization

#### X-ray photoelectron spectroscopy

The collected solid samples were analyzed using X-ray photoelectron spectroscopy (XPS) to obtain chemical characterization of the aerosols. For the XPS measurements a Perkin Elmer Phi 5500 multi technique system was used. The detailed setup of the machine during measurements was described in a previous work [20]. Commonly, the C 1s peak originating from the unavoidable atmospheric contamination is used as an internal standard for the binding energies (BEs) during XPS measurements. In the case of ruthenium the Ru 3d_5/2_ peak and the C 1s peak are overlapping, making this reference unreliable. To avoid this problem the gold foil conductively connected to the measured samples was used as an internal standard during the measurements. The experimental uncertainty of BE of the Ru 3d_5/2_ peak was determined to be ±0.1 eV. The curve fitting of the obtained spectra was made using PHI Multipak software (Ulvac-Phi, Inc.), assuming Shirley background. The asymmetrical shape of peaks was used due to the conductive nature of anhydrous RuO_2_ [21]. XPS analysis was performed using at least two different spots on the samples.

#### X-ray diffraction analysis (XRD)

Crystallographic structures of the collected aerosols were examined by XRD analysis. The combination of XPS and XRD analysis allowed the characterization of both crystalline and potentially amorphous compounds in the collected aerosols. XRD measurements were made using a Bruker D2 Phaser diffractometer with Cu Kα characteristic radiation, equipped with a scintillation detector. Rotation speed of the sample holder was 360°/min and the measurement angle interval was 20°–80° 2*θ*. The comparison of the obtained data with standards in the Joint Committee of Powder Diffraction Standards database [22] led to the crystal structure identification of the collected compounds.

## Results

### Release and transport results

#### Release of ruthenium

The amount of the released ruthenium from the crucible was obtained as the mass difference of the crucible with RuO_2_ precursor before and after the experiment. The obtained release rates are presented in Table 2.

**Table 2:** Release rates of ruthenium from the crucible

Experiment	Ruthenium release rate (mg/min)
(1) Air (1300 K)	0.34 ± 0.02
(2) Air (1500 K)	3.22 ± 0.16
(3) Air (1700 K)	20.27 ± 1.04

As can be seen from Table 2 the release rates of ruthenium increased with increased temperature. As the location of the injection of additional precursors into the airflow was just after the crucible, the precursors did not affect the vaporization of ruthenium and the observed ruthenium release results were as expected. When compared with the previous experiments the decrease in airflow over the crucible from 5.0 to 2.5 l/min resulted in an approx. 50% decrease of the ruthenium release rate from the crucible [7, 13]. This effect can be attributed to the lower absolute amount of oxygen available for the oxidation of ruthenium from the crucible.

#### Ruthenium transport

The quantities of transported ruthenium both in the form of aerosols and gaseous ruthenium trapped in sodium hydroxide traps were obtained by means of neutron activation and consequent gamma spectroscopy measurements. The obtained quantities, presented as % of released ruthenium, are shown in Table 3.

**Table 3:** Fractions of ruthenium transported as RuO_2_ aerosol particles and RuO_4_ gas through the model primary circuit and the fraction of ruthenium deposited inside the circuit

Exp. (#)	Ru transported in total (%)	RuO_2_ transported (%)	RuO_4_ transported (%)	Ru deposited (%)
(1) Air 1300 K	9.3 ± 0.9	9.1 ± 0.5	0.024 ± 0.012	90.7 ± 1.4
(2) Air 1500 K	12.8 ± 1.3	12.8 ± 0.6	0.010 ± 0.005	87.2 ± 1.9
(3) Air 1700 K	14.3 ± 1.4	14.3 ± 0.7	1E−4 ± 5E−5	85.7 ± 2.0
(4) NO_2_ 1300 K	13.9 ± 1.4	0.010 ± 0.005	13.9 ± 0.7	86.1 ± 2.0
(5) NO_2_ 1500 K	13.9 ± 1.4	4.0 ± 0.2	9.9 ± 0.5	86.1 ± 2.0
(6) NO_2_ 1700 K	20.2 ± 2.0	20.2 ± 1.0	2E−3 ± 1E−4	79.8 ± 3.1
(7) N_2_O 1300 K	6.1 ± 0.6	6.0 ± 0.3	0.13 ± 0.01	93.9 ± 1.0
(8) N_2_O 1500 K	25.5 ± 2.6	25.4 ± 1.7	0.14 ± 0.01	74.5 ± 3.8
(9) N_2_O 1700 K	15.5 ± 1.6	15.5 ± 0.8	0.001 ± 0.005	84.5 ± 2.3
(10) HNO_3_ 1300 K	10.4 ± 1.0	9.1 ± 0.5	1.2 ± 0.1	89.7 ± 1.6
(11) HNO_3_ 1500 K	13.1 ± 1.3	11.8 ± 0.6	1.3 ± 0.1	86.9 ± 2.0
(12) HNO_3_ 1700 K	14.4 ± 1.4	13.6 ± 0.7	0.78 ± 0.04	85.7 ± 2.2

The values are given as % of the released ruthenium. The uncertainties are stated as 2 standard deviations

After each experiment a significant amount of ruthenium was visually observed to be deposited at the outlet of the furnace, where the temperature gradient was the steepest. Similar behavior was observed in the previous work using the same facility [23]. The separate effects of different precursors are discussed in subsequent sections.

##### Air atmosphere

The masses of ruthenium transported in gaseous and aerosol forms in the humid air atmosphere were determined and are presented in Table 4. From Table 4 it can be seen that the aerosol form predominated over RuO_4_ over the entire temperature interval of the experiments (1300–1700 K). The increase of the temperature in the experiments decreased the transported fraction of gaseous ruthenium through the facility. This observation is in agreement with the trend in thermodynamic equilibrium calculations performed with the HSC 5.11 software [17] as well as with the trend presented in Fig. 1. The increased temperature in the experiments led to increased overall transport of ruthenium through the facility. When data from this work was compared with the previous experiments [13] the gaseous fraction of ruthenium transported through the facility was lower. This may indicate the effect of the flow rate on the transport of RuO_4_ through the RCS. A similar effect was observed in the study of Vér et al. [9] where very low flow rates were used.

**Table 4:** The mass of ruthenium transported as aerosol particles and as gas through the model primary circuit under a humid air atmosphere

Exp. (#)	Ru transported in total (mg)	Ru in the form of RuO_2_ aerosol (mg)	Ru in the form of RuO_4_ gas (mg)	Ratio of RuO_2_/RuO_4_	Ru deposited inside the facility (mg)
(1) 1300 K	0.64 ± 0.01	0.62 ± 0.001	0.020 ± 0.001	38 ± 1	8.4 ± 0.1
(2) 1500 K	8.3 ± 0.4	8.3 ± 0.4	0.010 ± 0.001	1636 ± 40	76.7 ± 0.8
(3) 1700 K	57.9 ± 2.9	57.9 ± 2.9	5E−4 ± 2.5E−5	1.25E5 ± 3.1E3	475.8 ± 4.8

The uncertainties are given as 2*σ* standard deviations

##### Atmosphere with 50 ppmV NO_2_

The results of ruthenium transport experiments under a humid air atmosphere with 50 ppmV of NO_2_ additive are shown in Table 5. The introduction of NO_2_ into the airflow had a significant effect on the composition of transported ruthenium. At temperatures of 1300 and 1500 K the fraction of the gaseous ruthenium transported through the facility was strongly increased when compared to the experiments in the humid air atmosphere at the same temperature.

**Table 5:** The mass of ruthenium transported as aerosol particles and as gas through the model primary circuit under a humid air atmosphere with 50 ppmV NO_2_

Exp. (#)	Ru transported in total (mg)	Ru in the form of RuO_2_ aerosol (mg)	Ru in the form of RuO_4_ gas (mg)	Ratio of RuO_2_/RuO_4_	Ru deposited inside the facility (mg)
(4) 1300 K	1.2 ± 0.1	0.001 ± 0.001	1.2 ± 0.1	0.0010 ± 0.0003	10.4 ± 0.1
(5) 1500 K	9.0 ± 0.5	2.6 ± 0.1	6.4 ± 0.3	0.40 ± 0.01	76.0 ± 2.2
(6) 1700 K	82.0 ± 4.1	82.0 ± 4.1	0.010 ± 0.005	13,231 ± 330	451.7 ± 8.3

The uncertainties are given as 2*σ* standard deviations

Over the entire experimental temperature range a strong increase of the transported gaseous ruthenium fraction and corresponding decrease of aerosol was observed when compared with the humid air atmosphere experiments. This behaviour can be explained by the oxidation of RuO_3_(g) in the hot zone of the furnace according to reaction (9). The equilibrium constants for reaction (9) were calculated using the HSC 5.11 software [17] and these values are presented in Table 6. The ratios between the transported aerosol and gaseous fractions of ruthenium as presented in Table 5 lower than thermodynamic equilibrium calculations predict (Table 6) in experiments 5 and 6 and higher in experiment 3.

9 $${\text{RuO}}_{3}({\text{g}}) + {\text{NO}}_{2}({\text{g}}) \leftrightarrow {\text{RuO}}_{4}({\text{g}}) + {\text{NO}}({\text{g}}).$$

**Table 6:** Equilibrium constants for the NO_2_ induced oxidation of RuO_3_ to RuO_4_ at different temperatures

Temperature (K)	*K*_eq_
1300	28.55
1500	16.85
1700	11.3

Additionally, as can be seen from Table 5, the fraction of gaseous ruthenium transported through the facility decreased with increasing temperature. This effect was attributed to two different phenomena; firstly the thermal decomposition of NO_2_ at high temperatures [24] according to reaction (10) [25]

10 $$2{\text{NO}}_{2}({\text{g}}) \leftrightarrow {\text{O}}_{2}({\text{g}}) + 2{\text{NO}}({\text{g}})\quad {\rm with}\;K = 1.5 \times 10^{13} \exp (- 65,400/RT)\;{\text{mol}}^{- 1} \;{\text{s}}^{- 1},$$

and secondly the decreasing ability of NO_2_ to oxidize RuO_3_(g) to RuO_4_(g) as presented in reaction (9), with a temperature increase according to the equilibrium constants presented in Table 6.

As can be seen from the data in Tables 4 and 5, the total amount of transported ruthenium increased over the entire temperature range when compared to the humid air atmosphere.

##### Atmosphere with 50 ppmV N_2_O

The obtained amounts of ruthenium transported through the facility with injection of N_2_O gas are presented in Table 7. The injection of N_2_O increased the transported aerosol fraction of ruthenium when compared to the humid air experiments. This behavior was partly attributed to reactions (11) and (12) and the subsequent decomposition of RuO_3_ into solid RuO_2_ at the outlet of the hot zone from the furnace, where a temperature decrease below 1000 K was observed.

11 $${\text{RuO}}_{4}({\text{g}}) + {\text{N}}_{2}{\text{O}}({\text{g}}) \leftrightarrow {\text{RuO}}_{3}({\text{g}}) + 2{\text{NO}}({\text{g}}),$$

12 $${\text{RuO}}_{4}({\text{g}}) + 2{\text{N}}_{2}{\text{O}}({\text{g}}) \leftrightarrow {\text{RuO}}_{2}({\text{s}}) + 4{\text{NO}}({\text{g}}).$$

**Table 7:** Mass of ruthenium transported as aerosol particles and as gas through the model primary circuit under a humid air atmosphere with 50 ppmV N_2_O

Exp. (#)	Ru transported in total (mg)	Ru in the form of RuO_2_ aerosol (mg)	Ru in the form of RuO_4_ gas (mg)	Ratio of RuO_2_/RuO_4_	Ru deposited inside the facility (mg)
(7) 1300 K	0.50 ± 0.03	0.50 ± 0.03	0.010 ± 0.005	47.0 ± 1.2	11.1 ± 0.1
(8) 1500 K	16.5 ± 0.8	16.4 ± 0.8	0.090 ± 0.005	177 ± 4.4	68.5 ± 0.9
(9) 1700 K	62.9 ± 3.1	62.9 ± 3.1	0.010 ± 0.005	6123 ± 153	470.8 ± 3.1

The uncertainties are stated as 2*σ* standard deviations

The amount of total ruthenium transported showed strong temperature dependence behavior. At 1300 K there was a decrease in the total amount of transported ruthenium in comparison to the humid air atmosphere. At 1500 K the total amount of transported ruthenium was almost double that observed in humid air. At 1700 K the observed increase of ruthenium transport due to NO_2_ injection was statistically insignificant when compared to the humid air experiments.

##### Atmosphere with 5 ppmV HNO_3_

The quantities of ruthenium transported in an atmosphere with 5 ppmV HNO_3_ are presented in Table 8. The introduction of HNO_3_ into the airflow resulted in a higher gaseous fraction of ruthenium being transported through the facility when compared to the humid air atmosphere. This effect was observed over the entire temperature range used in the experiments.

**Table 8:** Mass of ruthenium transported as aerosol particles and as gas through the model primary circuit under a humid air atmosphere with 5 ppmV HNO_3_

Exp. (#)	Ru transported in total (mg)	Ru in the form of RuO_2_ aerosol (mg)	Ru in the form of RuO_4_ gas (mg)	Ratio of RuO_2_/RuO_4_	Ru deposited inside the facility (mg)
(10) 1300 K	0.9 ± 0.5	0.80 ± 0.04	0.11 ± 0.01	7.5 ± 0.2	10.7 ± 0.5
(11) 1500 K	8.5 ± 0.4	7.6 ± 0.4	0.86 ± 0.04	8.8 ± 0.2	76.5 ± 0.5
(12) 1700 K	58.2 ± 3.0	55.0 ± 2.8	3.2 ± 0.2	17.5 ± 0.4	475.5 ± 3.0

The uncertainties are stated as 2*σ* standard deviations

As can be seen from Tables 3 and 8 the effect of nitric acid was not as prominent as predicted by the thermodynamic calculations. These, calculated using HSC 5.11 software, indicated that *K* values for reaction (13) would be 1.65E11, 4.57E10 and 1.66E10 for temperatures 1300, 1500 and 1700 K, respectively [17]. This observation can again be explained by the thermal decomposition of HNO_3_ to the lower nitrogen oxides [26, 27] at elevated temperatures, thus lowering the amount of precursor in the gas phase.

13 $$3{\text{RuO}}_{3}({\text{g}}) + 2{\text{HNO}}_{3}({\text{g}}) \leftrightarrow 3{\text{RuO}}_{4}({\text{g}}) + {\text{H}}_{2}{\text{O}}({\text{g}}) + 2{\text{NO}}({\text{g}}).$$

When the values in Tables 4 and 8 were compared, the total amount of transported ruthenium was fairly similar when compared to the humid air atmosphere over the entire temperature range used in the experiments.

#### Online monitoring of aerosol transport

**Fig. 3:**
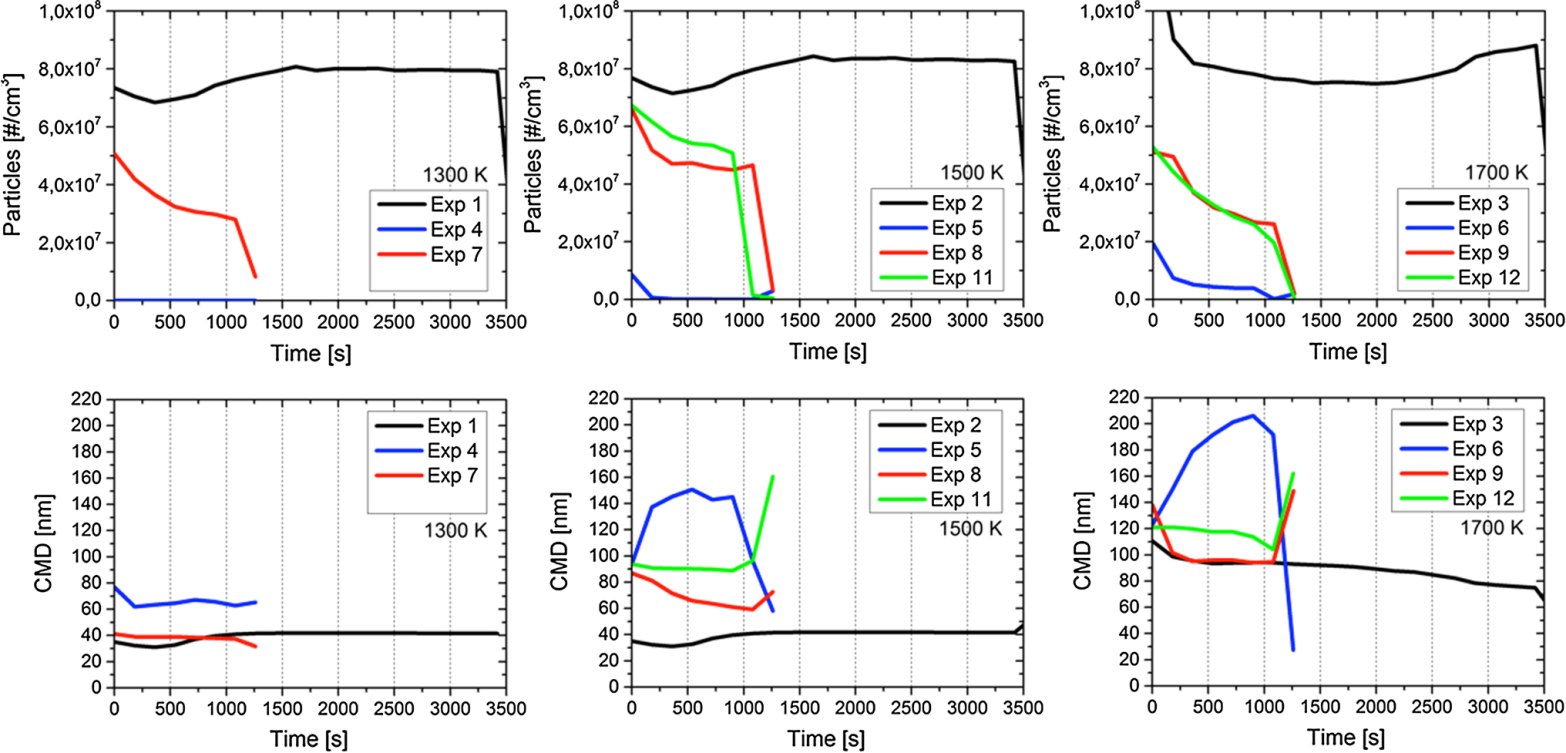
The particle number concentration (#/cm^3^, *above*) and count median diameter (nm, *below*) at the outlet of the facility during the experiments (measured with SMPS). The duration of experiments *1*–*3* was 60 min, whereas the other experiments lasted for 20 min

**Fig. 4:**
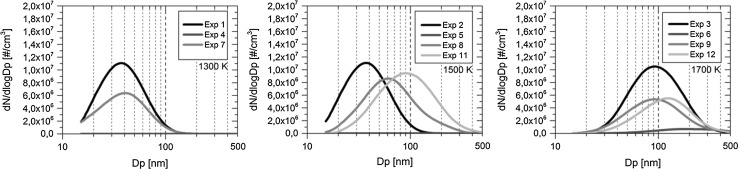
The particle number size distribution at the time point of 750 s since the beginning of each experiment (measured with SMPS)

In order to understand the transient behavior of ruthenium, the transport of aerosol particles through the facility was followed online. The number concentration, diameter and number size distribution of particles were measured with SMPS at the outlet of the facility. The range of measurement uncertainty (±10% in the experiments) is not displayed in Fig. 3 or 4. The data for experiment 10 is not presented as there was a fault in the online measurement.

In Fig. 3, the development of particle number concentration and the count median diameter (CMD) of particles over the course of the experiments are presented. On the basis of the measurement results, the number concentration of particles remained at a rather similar level in the experiments when only the effect of RuO_2_ vaporization temperature was examined. However, the effect of vaporization temperature on the diameter of particles was significant. The temperature increase from 1300 to 1700 K caused an increase in particle diameter in every experiment, resulting in up to 3.5 times larger particles in the case of NO_2_ feed. This phenomenon is directly connected to a higher release of ruthenium from the crucible and to the subsequent formation of particles. High release of ruthenium also favors the agglomeration of particles when the concentration of particles exceeds ca. 10^6^ particles/cm^3^ [28].

The transport of particles was affected by the feed of nitrogen compounds (NO_2_, N_2_O, HNO_3_) into the flow of Ru oxides when compared with reference experiments 1–3. In general, the number concentration of particles decreased, but at the same time the diameter of particles seemed to increase. Depending on the experiment, the particle CMD ranged from ca. 20 to 210 nm. In the case of NO_2_ feed, the measured particle concentration was at the lowest level, ranging mainly from ca. 10^3^ to 10^6^ particles/cm^3^ in experiments 4 and 5. The concentration increased in experiment 6 and was observed to be between ca. 10^6^ and 10^7^ particles/cm^3^. Furthermore, the particle diameter was also greatest in experiment 6 and seemed to even increase strongly over the course of the experiment. This indicates, in addition to the agglomeration of particles, that part of the formed gaseous Ru compounds were probably condensing on the surface of the existing particles, thereby increasing the particle diameter. This conclusion is also supported by the low number concentration of particles measured and the previous observation of high formation of gaseous Ru due to NO_2_, see [13]. The effects of N_2_O and HNO_3_ feeds on the particle properties were not as strong as the NO_2_ feed. Therefore, the observed effects on the particle number concentration and particle diameter were in between the range limited by the reference experiments and NO_2_ experiments (see above).

The particle number size distribution for a particle diameter range from 15 to 500 nm in the experiments is presented in Fig. 4. The data is presented at the time point of 750 s since the beginning of each experiment. In addition to the above observations on particle behavior, it was noticed that the transported particles were lognormally distributed and that most of the particles were smaller than 500 nm in diameter. The feed of nitrogen compounds N_2_O and HNO_3_ under the studied conditions did not vary the shape of the particle number size distribution greatly. The broad particle distribution and the predominance of large particles (100–500 nm) in the distribution were evident when NO_2_ was present in the atmosphere, see for example the case of 1700 K.

### Chemical characterization

#### XPS analysis

The aerosol particles transported through the facility were collected on PTFE filters and then examined with XPS. With the XPS technique the BEs of electrons in the elements of interest could be determined. The identification of chemical composition was obtained by comparing determined BE values with the reference values from the literature. In the cases of anhydrous and hydrated RuO_2_ references commercial powders (purity 99.5%, Alfa Aesar) were analyzed in-house and the obtained reference spectra were then compared with the spectra of the collected aerosols.

A selection of the reference BE values used during the evaluation are presented in Table 9. From the values in Table 9 it is clear that the BE is not only dependent on the oxidation state of ruthenium but also on the chemical environment, e.g., the hydration of RuO_2_. Similar observations were also made in a previous study [29].

**Table 9:** Reference values for the electron binding energies of various ruthenium compounds

Compounds	Binding energy of Ru 3d_5/2_ line (eV)
RuO_2_	280.5 [20]
RuO_2_·H_2_O	282.1 [20]
RuO_4_	283.3 [32]
BaRuO_4_	284.2 [33]
RuCl_3_	282.1 [33]
Ru (metal)	280.0 [32]

**Fig. 5:**
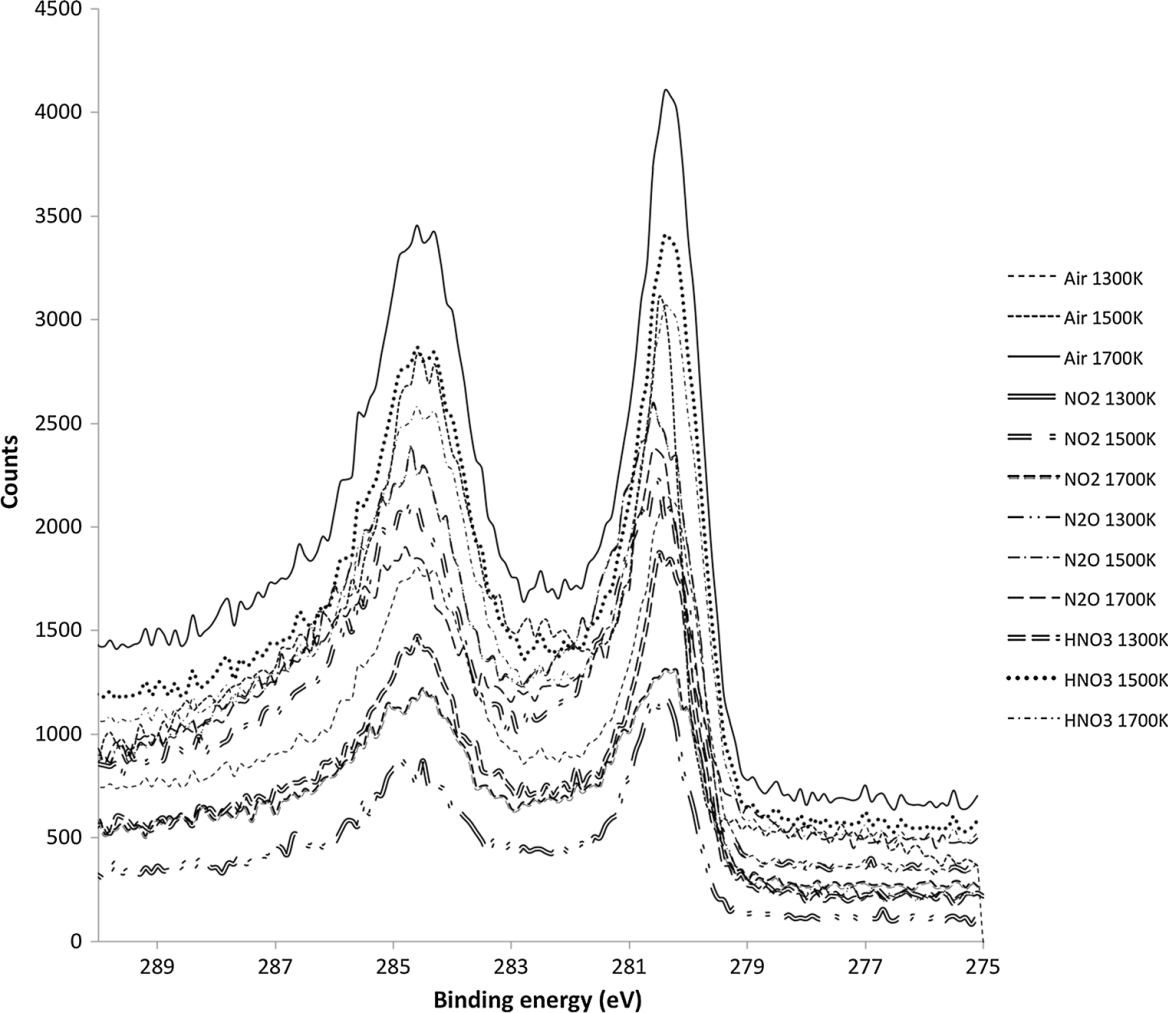
The XPS spectra obtained from measurements of aerosols collected on PTFE filters. Spectra were scaled to fit the figure

The BEs of the Ru 3d_5/2_ peak in all samples were determined to be within the interval of 280.4–280.5 eV, as presented in Fig. 5. This value provides a very good fit with the Ru 3d_5/2_ BE in the anhydrous form of RuO_2_, thus indicating that the form of ruthenium in the transported aerosol was anhydrous RuO_2_ under all experimental conditions. The overall characteristics of the spectra are very similar to each other, therefore strengthening the assumption that all obtained spectra originate from the same compound.

Nitrogen was not detected in the collected aerosol samples. The possible formation of ruthenium nitrosyl compounds [30, 31] could therefore be ruled out during the data evaluation process.

#### XRD

**Fig. 6:**
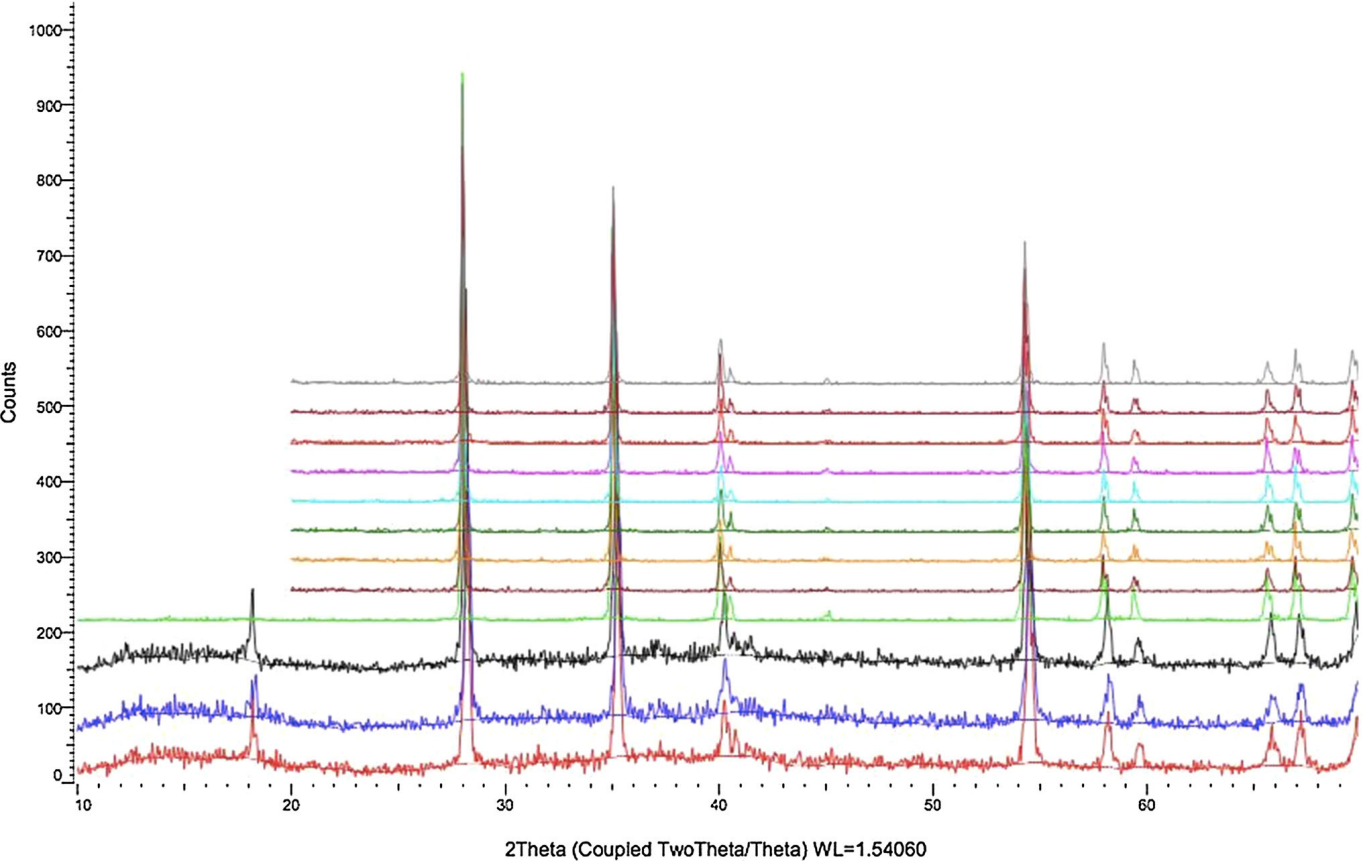
The XRD spectra obtained from the samples in experiments *1*–*12*. The height of the peaks was scaled in order to fit in the figure

The spectra obtained from the qualitative crystallographic XRD analysis of the collected aerosol samples are presented in Fig. 6. The XRD spectra recorded from experiments 1 to 12 showed the same diffraction pattern, which corresponds to the rutile structure of RuO_2_. This is in good agreement with the XPS analysis, leading to the conclusion that aerosols collected from the gas flow were in the form of anhydrous ruthenium dioxide.

## Conclusions

The main objective of this study was to evaluate the effects of different nitrogen compounds on the transport and chemical composition of ruthenium in a model primary circuit of NPP. Nitrogen oxides and HNO_3_ represented the air radiolysis products unavoidably formed during a nuclear accident connected with air ingress sequence. The experiments were performed at temperatures of 1300, 1500 and 1700 K, with air atmosphere to simulate an air ingress type of accident. The examination of the quantities and chemical composition of transported ruthenium both in aerosol and gaseous form through the primary circuit simulating facility was within the scope of the study.

The effects of humid air, NO_2_, N_2_O and HNO_3_ on the transport and partitioning of ruthenium were investigated in this work.

It was shown that the release rate of ruthenium (given as for elemental Ru) from the ruthenium dioxide powder was strongly dependent on the temperature of the experiment. The determined release rate values were 0.34 ± 0.07 mg/min at 1300 K, 3.22 ± 0.16 mg/min at 1500 K and 20.27 ± 1.04 mg/min at 1700 K in an air atmosphere with a low steam content (≈2.1E4 ppmV).

The quantification and partitioning of ruthenium was obtained by collection of aerosols on PTFE filters and trapping of the gaseous fraction in 1 M NaOH solution. It was visually observed that the major part of the released ruthenium was deposited within the area of the furnace outlet where the temperature gradient was the steepest. It was also shown that the temperature increase during the experiments increased not only the release but also the transport of ruthenium through the facility. The quantification of ruthenium transport demonstrated a significant impact of the gaseous additives on both the absolute amount and on the partitioning of the transported ruthenium between gaseous and aerosol fractions.

Addition of NO_2_ in a concentration of 50 ppmV into the gas stream significantly increased the gaseous fraction of ruthenium transported through the facility at all experimental temperatures. The overall transport of ruthenium was increased when compared to the humid air atmosphere at 1300 and 1700 K when NO_2_ was injected into the gas stream.

The number concentration of particles at the outlet of the facility was low in the case of NO_2_ feed, but the diameter of particles seemed to increase over the course of the experiments. These observations indicated the likelihood that part of the formed gaseous Ru compounds condensed on the surface of the existing particles and thereby increased the particle diameter.

Introduction of 50 ppmV N_2_O into the gas phase led to an increased fraction of ruthenium transported in the form of aerosols. The gaseous fraction of transported ruthenium was increased under all experimental temperatures. A very significant (almost 100%) increase in total transported ruthenium was observed in the experiment conducted at 1500 K when compared to the humid air atmosphere experiments.

With the injection of 5 ppmV HNO_3_ into the gas stream the transport of gaseous ruthenium increased at all studied temperatures. The overall transport of ruthenium with HNO_3_ in the air-flow was similar to that observed in the humid air atmosphere.

The examination of aerosols collected from the experiments by means of XPS and XRD techniques showed the same chemical speciation (anhydrous RuO_2_) over the entire range of experimental conditions.

The results obtained in this study showed a significant effect of nitrogen compounds on the transport of ruthenium in the primary circuit conditions. Introduction of nitrogen oxides and nitric acid into the gas stream promoted the transport of ruthenium tetroxide through the primary circuit simulating facility. The data obtained during this study provide additional insight into the ruthenium chemistry during a nuclear accident and reveal the possible interactions of ruthenium with air radiolysis products.
